# Human Short Peptidoglycan Recognition Protein PGLYRP1/Tag-7/PGRP-S Inhibits *Listeria monocytogenes* Intracellular Survival in Macrophages

**DOI:** 10.3389/fcimb.2020.582803

**Published:** 2020-12-23

**Authors:** Darya Slonova, Alexandra Posvyatenko, Alexey Kibardin, Elena Sysolyatina, Elena Lyssuk, Svetlana Ermolaeva, Sergei Obydennyi, Nikolay Gnuchev, Georgii Georgiev, Konstantin Severinov, Sergey Larin

**Affiliations:** ^1^ Laboratory of Molecular Microbiology, Center of Life Sciences, Skolkovo Institute of Science and Technology, Moscow, Russia; ^2^ Laboratory of Molecular Immunology, Dmitry Rogachev National Medical Research Center of Pediatric Hematology, Oncology and Immunology, Russian Ministry of Health, Moscow, Russia; ^3^ Laboratory of Ecology of Pathogenic Bacteria, Gamaleya National Research Centre of Epidemiology and Microbiology, Russian Ministry of Health, Moscow, Russia; ^4^ Laboratory of Intracellular Signaling and Systems Biology, Centre for Theoretical Problems of Physicochemical Pharmacology, Moscow, Russia; ^5^ Laboratory of Cellular Hemostasis and Thrombosis, Dmitry Rogachev National Medical Research Center Of Pediatric Hematology, Oncology and Immunology, Russian Ministry of Health, Moscow, Russia; ^6^ Laboratory of Cancer Immunogenetics, Institute of Gene Biology, Russian Academy of Sciences, Moscow, Russia

**Keywords:** PGLYRP1 protein, Tag-7, peptidoglycan recognition protein-S, *Listeria monocytogenes*, peptidoglycan recognition protein, phagocytosis, innate immunity

## Abstract

PGLYRP1/Tag-7/PGRP-S is one of mammalian peptidoglycan recognition proteins (PGRPs). Here, we demonstrate that human recombinant PGLYRP1/Tag-7/PGRP-S potentiates the response of murine macrophage-like ANA-1 cells and human macrophages to facultative intracellular pathogen *Listeria monocytogenes*. PGLYRP1/Tag-7/PGRP-S binds to the surface of *L. monocytogenes* and other bacterial cells but has no effect on their growth in culture. While PGLYRP1/Tag-7/PGRP-S treatment modestly enhanced phagocytosis of bacteria by ANA-1 cells, the intracellular survival of PGLYRP1/Tag-7/PGRP-S treated *L. monocytogenes* was strongly inhibited 2 h after internalization. PGLYRP1/Tag-7/PGRP-S treatment of bacteria boosted oxidative burst induction and increased the level of proinflammatory cytokine IL-6 produced by ANA-1, however, these effects happened too late to be responsible for decreased intracellular survival of bacteria. Our results thus suggest that PGLYRP1/Tag-7/PGRP-S acts as a molecular sensor for detection of *L. monocytogenes* infection of mammalian cells that leads to increased killing through a mechanism(s) that remains to be defined.

## Introduction

Mammalian immunity can be subdivided into innate immunity and adaptive, or specific, immunity (Rodríguez et al., 2012). Evolutionarily, the innate immune system is a more ancient part of the host defense against infection. Innate immunity uses various response mechanisms, such as recognition of pathogens and killing by dedicated cells (e.g., macrophages engulf bacteria and produce several substances for their killing, including reactive oxygen species), and presentation of specific parts of the pathogen to adaptive immunity (Medzhitov et al., 1997).

Innate immunity recognizes characteristic conserved structures of microorganisms which are absent from the host and are called pathogen-associated molecular patterns (PAMPs) (Janeway, 1989). PAMPs include lipopolysaccharides of Gram-negative bacteria, lipoteichoic acids of Gram-positive bacteria, bacterial DNA and others. Peptidoglycan, which is an essential and specific component of almost all bacteria (Rogers et al., 1980), also serves as a target recognized by innate immunity.

Eukaryotes possess several types of receptors, which recognize peptidoglycan. One of them is a family of peptidoglycan recognition proteins (PGRPs) (Dziarski, 2003). In insects there are many peptidoglycan recognition proteins (for example, *Drosophila melanogaster* has 19, whereas *Anopheles gambiae* has nine (Dziarski et al., 2006)), but mammals have only four PGRPs: PGRP-S/Tag-7/PGLYRP1 (further referred to as PGLYRP1) (Kustikova et al., 1996; Kang et al., 1998), PGRP-L/Tag-L/PGLYRP2 (Liu et al., 2001; Kibardin et al., 2003), PGRP-Iα/PGLYRP3 (Liu et al., 2001), and PGRP-Iβ/PGLYRP4 (Liu et al., 2001), named so for the length of their transcripts (“S” - short, “L” - long, and “I” - intermediate. PGLYRP1(current designation) was independently identified as Tag-7, a protein capable of inducing potent mouse immune response (Kustikova et al., 1996) and as 19-kDa protein PGRP-S, which activated the prophenoloxidase cascade, an antimicrobial mechanism of host defense in insects (Yoshida et al., 1996). Steiner’s group showed that PGRPs were members of a highly conserved family (Kang et al., 1998). PGLYRP1 can be secreted outside the cells and may interact with other components of innate immunity system by paracrine signaling.

Listeriosis is a severe foodborne infection caused by the Gram-positive bacterium *Listeria monocytogenes* (Vázquez-boland et al., 2001; Wing and Gregory, 2002). In humans, the major clinical signs of listeriosis are sepsis, meningitis, and meningoencephalitis (Allerberger and Wagner, 2010). *L. monocytogenes* is a facultative intracellular pathogen, which has developed a number of special mechanisms to escape the innate immune response (Calame et al., 2016). The intracellular life cycle of *L. monocytogenes* includes entrance into the host cell *via* phagocytosis, lysis of the phagosomal membrane, and escape into the cytosol, where it replicates freely. Intra- and inter-cellular spreading of the pathogen is accomplished *via* protrusions of infected cell membrane that form when the pathogen harnesses host cell actin cytoskeleton (Calame et al., 2016). Previously we have shown that PGLYRP2/Tag-L/PGRP-L, when overexpressed in human colon adenocarcinoma HT29 cells, impaired bacterial invasion and early intracellular growth by decreasing the viability of intraphagosomal bacteria (Kibardin et al., 2006). Here, we examined the effects of secreted PGLYRP1 on *L. monocytogenes* interactions with eukaryotic cells. We demonstrate that PGLYRP1 protects mouse macrophages from *L. monocytogenes* infection by strongly enhancing intracellular bacterial killing. PGLYRP1 also potentiates proinflammatory response in mouse macrophages after bacterial exposure, however this effect is likely unrelated to increased killing of internalized bacteria.

## Materials and Methods

### Cell Lines and Growth Conditions

ANA-1 (murine macrophage cell line) cells, originally purchased from ATCC, were cultured in clear plastic TC-treated dishes or multiple-well plates (Corning Inc.) in a HeraCell 150 incubator (Thermo Electron Corp.) at 37°C, 95% humidity and 5% CO_2_. Dulbecco’s modified Eagle’s medium (DMEM, HyClone) was supplemented with 10% fetal bovine serum, 2 mM L-glutamine (all from HyClone), 100 un/ml penicillin (Gibco), and 100 µg/ml streptomycin (OJSC Biokhimik, Russia).

CHO-S cells were cultivated in CD OptiCHO medium (Gibco) supplemented with 4 mM L-glutamine (HyClone) and 0.18% Pluronic F-68 (Gibco) at 37°C, 5% CO_2_. Cells were transfected by electroporation with the PGLYRP1 expression construct. Stable clones were generated by positive selection with 500 µg/ml G418 (Calbiochem). The clone with the highest PGLYRP1 production level was used for preparation of conditioned medium.

Human macrophages were differentiated from peripheral blood monocytes. Monocytes were isolated from three healthy donors using Ficoll-Paque Premium (GE Healthcare) and adherence method (Fuss et al., 2009). Monocytes were incubated in RPMI-1640 medium supplemented with 2% heat-inactivated human AB serum, 2 mM L-glutamine, 10 mM HEPES, 50 µM β-mercaptoethanol, 2 mM sodium pyruvate, and 2 mM MEM Vitamin (HyClone) at 37°C, 5% CO_2_ for 6 days. On the 4th day, the media was fully refreshed. On the first and the fourth days 50 ng/ml GM-CSF (Sci-Store) was added to the media. Cells were stained with fluorophore-conjugated primary antibodies against CD11b (APC-Cy7), CD80 (PE-Cy5), CD86 (BV421), HLA-DR (PE-Cy7) and analyzed by flow cytometer (Navios, Beckman Coulter, Inc.).

### Bacterial Strains

*Staphylococcus aureus*, *Streptococcus pneumoniae*, and *Escherichia coli* strains used in this work were clinical isolates from the Russian Ministry of Health Dmitry Rogachev National Medical Research Center Of Pediatric Hematology, Oncology and Immunology collection. *Listeria monocytogenes* type strain EGDe (serovar 1/2a) was cultivated in the Brain Heart Infusion medium (BHI, BD, USA) at 37°C.

For fixation, bacteria from overnight cultures were precipitated by centrifugation for 5 min at 5000 rpm; the pellet was resuspended in PBS, bacteria were precipitated again, resuspended in 2.5% glutaraldehyde in PBS and incubated for 2 h at 4°C. The final concentration of bacteria was 10^9^ cells/ml. Fixed or live *L. monocytogenes* cells were labelled with Cy3 (Mono-Reactive Dye Pack, GE Healthcare, Sweden) in accordance with the recommendations of the manufacturer.

### PGLYRP1 Purification

CHO-S cells stably transfected to express PGLYRP1 tagged with the DED epitope (patent RU2380373) were cultivated in CD OptiCHO medium (Gibco) with shaking at a density of ~15 × 10^6^ cells per ml for 4 days. Cells were removed by centrifugation and the culture supernatant was filtered through a 0.22 PES filter (Corning). Cleared supernatant was loaded by gravity flow onto a column of CL6B Sepharose (GE Healthcare, Sweden) conjugated with antiDED-specific mAb (Proteinsynthesis, Moscow, Russia). All chromatographic steps were performed at 4°C. The column was washed with 5 volumes of 20 mM sodium phosphate (pH 7.5) buffer with 150 mM NaCl and then with 5 volumes of same buffer with 1 M NaCl. PGLYRP1 was eluted with 20 mM sodium citrate (pH 2.5) buffer. Next, pH was adjusted to 7.5 by dropwise addition of 0.1 M NaOH. Fractions with PGLYRP1 were subjected to dialysis against 10 mM sodium phosphate (pH 7.5) buffer with 150 mM NaCl. Purity of PGLYRP1 was assessed to be ~98% by Coomassie staining of SDS gels and volumetric analysis (Quantity One software for ChemiDoc XRS system). The concentration of pure protein was determined by the bicinchoninic acid assay (Sigma).

### Binding Assay

The equal amount of dead *Staphylococcus aureus*, *Streptococcus pneumoniae* and *Escherichia coli* from clinical isolates and various amount of dead *L. monocytogenes* (10^8^, 10^7^, 10^6^, 10^5^, 10^4^) were resuspended in 30 µl of PBS with PGLYRP1 (40 µg/ml). After 1 h incubation at room temperature (RT), the bacterial suspensions were centrifuged, supernatants from *L. monocytogenes* were saved, all cells were washed and resuspended in PBS. Bacteria incubated in the absence of PGLYRP1 were used as control. The pellets (for all cultures) and supernatants (only for *L. monocytogenes*) were subjected to SDS-PAGE and immunoblotting analysis with polyclonal antibodies against recombinant human PGLYRP1 (1:1000) and horseradish peroxidase-conjugated mouse antibodies against mouse Ig (1:5000). The antibodies were diluted with the blocking buffer TBS-T (0.05% Tween 20 (Sigma) in TBS (Sigma)), 3% fat-free milk. The samples were resolved by SDS-PAGE and blotted onto a PVDF membrane HYBOND-P (GE Healthcare, UK). The blots were developed using a detection system Immobilon Western (Millipore, USA), in accordance with the recommendations of the manufacturer.

### Bacterial Phagocytosis

Cy3-labelled *L. monocytogenes* were pre-incubated with PGLYRP1 or in the growth medium alone for 1 h at RT. ANA-1 cells were incubated with bacteria (~5 bacteria/cell) and 5 µg/ml of PGLYRP1. After 1 h at 37°C, ANA-1 cells were washed with PBS and detached with trypsin (HyClone). After trypsin neutralization by growth medium, cells were harvested by centrifugation (5 min at 1000 rpm), and the pellet was washed 3 times and resuspended in PBS. The suspensions were analyzed by a flow cytometer (Cytomics FC 500 MPL, Beckman Coulter, Inc.) to determine uptake of the Cy3 bacteria. All assays were done in DMEM supplemented with FBS, glutamine and penicillin/streptomycin.

### *In Vitro L. monocytogenes* Invasion and Proliferation Assay

The invasion assay was performed as described (Kibardin et al., 2006). ANA-1 cells were grown on 24-well plate to density 200 000 cells per well. Bacteria from exponential cultures (OD_600nm_≈1.5) were kept frozen in 10% glycerin and thawed on the day of the experiment. 0.5 ml bacterial suspension was diluted in pre-warmed DMEM so that the multiplicity of infection (MOI) was 100 CFU per cell and added to ANA-1. After 1 h incubation at 37°C, the cells were extensively washed, and the medium was changed to fresh DMEM supplemented with 100 µg/ml gentamicin (Sigma) to kill extracellular bacteria. The number of successfully entered bacteria was assessed after 1 h incubation with gentamicin by plating serial dilutions of cell lysates obtained after 5 min incubation with 1% Triton X 100 (Sigma). The effectiveness of intracellular proliferation was evaluated by a number of CFU of the entered bacteria.

### Analysis of Intracellular Localization of *L. monocytogenes* by Confocal Microscopy

ANA-1 cells were incubated on sterile glass slides inside 6-well plates (total 100 000 cells per glass slide) for 4 h at 37**°**C, 95% humidity and 5% CO_2_ for adhesion. Cells were DAPI (Sigma) stained. Internalization assays were performed as described above using Cy3-labelled live *L. monocytogenes*. Images were acquired using an Axio Observer Z1microscope (Carl Zeiss, Jena, Germany) equipped with a Yokogawa spinning disc confocal device (CSU-X1; Yokogawa Corporation of America, Sugar Land, TX) and a 1.3 numerical aperture ×100 objective.

### Oxidative Burst Assay

ANA-1 cells were incubated in a 96-well white plate (Greiner) (200 000 cells per well) at 37°C, 5% CO_2_. The cells were incubated for 1 h in the presence of PGLYRP1 or albumin in 45 µl of the DMEM (phenol red-free, Gibco) along. For oxidative burst detection luminol was used. 25 mM PMA (phorbol-12-myristate-13-acetate) or fixed *L. monocytogenes* (~100 bacteria per cell) were used as stimulus. The chemiluminescence was detected by CLARIOstar (BMG LABTECH). For standard curve calculations, the MARS Data analysis software was used.

### Cytokine Secretion Assay

ANA-1 cells were incubated with the proteins (5 µg/ml of PGLYRP1, albumin) or PBS as control in the presence or absence of dead *L. monocytogenes* (~100 bacteria per cell) or alive *L. monocytogenes* (~5 bacteria per cell). After 48 h incubation, the culture supernatant was analyzed by ELISA (DuoSet Mouse IL-6 R&D Systems, Oxon, GB), in accordance with the recommendations of the manufacturer.

### Statistical Analysis

Each experiment was done in three replicates. The statistical significance of the results was analyzed using the Mann-Whitney U-test (*p ≤ 0.05).

## Results

### Recombinant Human PGLYRP1 Produced in Eukaryotic Expression System Forms Homodimers

**Figure 1:**
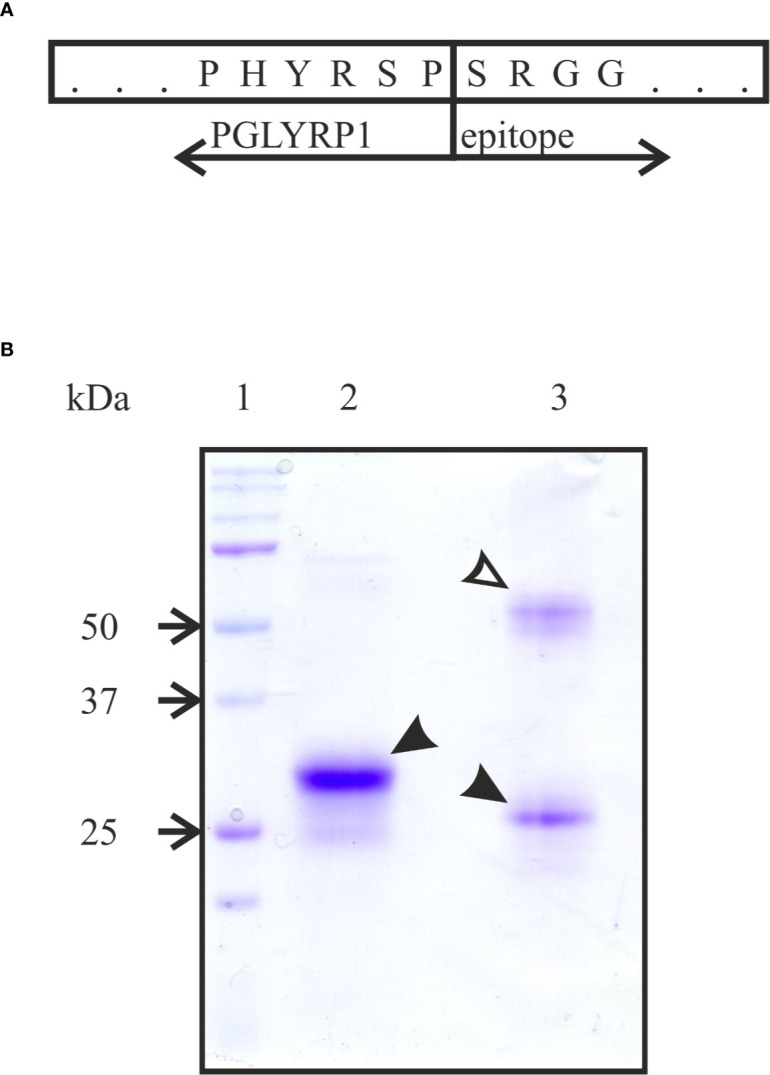
Purification of recombinant tagged PGLYRP1. **(A)** A scheme of the fusion of full-sized PGLYRP1 to the triple DED epitope. The last 6 amino acids of PGLYRP1 and the 4 first amino acids of the DED epitope are shown. **(B)** Recombinant PGLYRP1 (3 µg) purified as described in the *Materials and Methods* section was subjected to SDS gel electrophoresis. In lane 2, the loading Laemmli buffer contained a reducing agent (5 mM β-mercaptoethanol). In lane 3, the loading buffer without β-mercaptoethanol was used. Lane 1 is a marker lane. A Coomassie stained gel is presented. At reducing conditions, the recombinant PGLYRP1 migrates as a single 30 kDa band (black arrow). At non-reducing conditions, PGLYRP1 migrates as two bands likely corresponding to a homodimer (~60 kDa, open arrow) and a monomer (25 kDa, black arrow).

To produce human recombinant PGLYRP1, cDNA of human PGLYRP1 was fused with DNA coding for a C-terminal triple DED epitope (**Figure 1A**). The CHO-S cell line was stably transfected with the resulting construct and clones expressing recombinant PGLYRP1 were isolated. The recombinant protein was purified from cultured medium by immunoaffinity chromatography with antibodies against the DED epitope. Analysis of purified protein by SDS gel electrophoresis demonstrated that in the presence of reducing agent (5 mM β-mercaptoethanol) in the loading buffer, the protein migrated as a single band with molecular mass of approximately 30 kDa (**Figure 1B**, lane 2). In the absence of the reducing agent, two bands of similar intensity with apparent molecular weights of ~25 and ~60 kDa were observed (**Figure 1B**, lane 3). Both bands contained PGLYRP1 based on MALDI-MS analysis. Previously, dimerization of native PGLYRP1 through formation of disulfide bonds was reported (Kiselev et al., 1998; Lu et al., 2006). Apparently, recombinant PGLYRP1 also forms homodimers. The difference in electrophoretic mobility of bands corresponding to monomeric PGLYRP1 in the presence and in the absence of reducing agent suggests that additional disulfide bonds may form within the monomer.

### Recombinant Human PGLYRP1 Binds to Gram-Positive and Gram-Negative Bacteria Cell Walls

**Figure 2:**
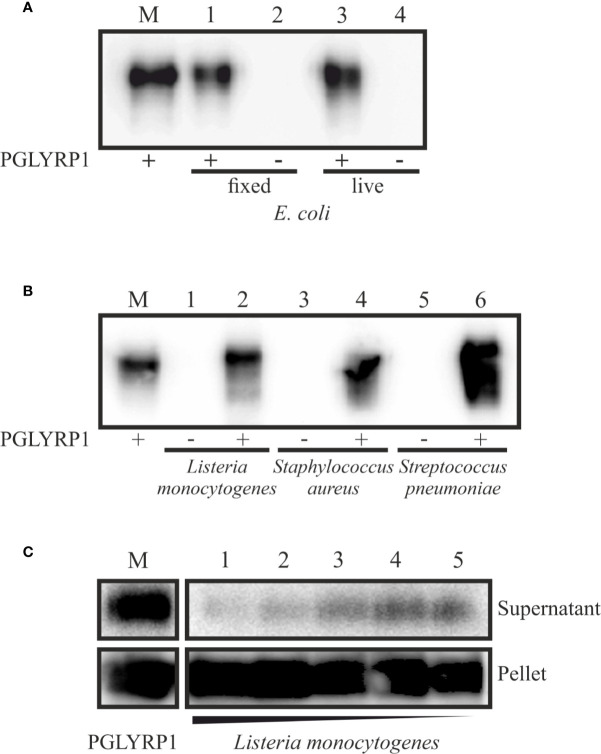
Recombinant PGLYRP1 binds to both Gram-positive and Gram-negative bacteria cells. The recombinant PGLYRP1 was incubated for 1 h at 25°C with 1.8∙10^7^ of live or fixed bacteria. Bacteria were pelleted by centrifugation and the presence of PGLYRP1 in the pellets was determined by SDS PAGE followed by a Western blot with anti-PGLYRP1 antibodies. In all panels, the amount of protein initially added to bacterial cells is shown in lane M for comparison with bound protein amounts. **(A)** Binding of PGLYRP1 to living (lane 1) or fixed (lane 3) *E. coli* cells. Lanes 2 and 4 are controls (no PGLYRP1 added to bacteria). **(B)** Binding of PGLYRP1 to fixed *Listeria monocytogenes* (lane 2), *Staphylococcus aureus* (lane 4), and *Streptococcus pneumoniae* (lane 6). **(C)** Binding of PGLYRP1 to decreasing amounts of fixed *Listeria monocytogenes* cells. PGLYRP1 was combined with 10^8^ (lane 1), 10^7^ (lane 2), 10^6^ (lane 3), 10^5^ (lane 4), or 10^4^ (lane 5) of fixed *Listeria monocytogenes* cells. Cells were collected by centrifugation and partition of PGLYRP1 between the pellets and supernatants was determined. The amount of protein initially added to bacterial cells is shown in lane M.

To verify whether recombinant PGLYRP1 interacts with bacterial cell walls, a binding assay was performed. PGLYRP1 was incubated with suspensions of live *E. coli* or cells fixed with glutaraldehyde (the effect of glutaraldehyde fixation was tested since some experiments with *L. monocytogenes* described below required fixation). After low speed centrifugation, bacterial pellets were extensively washed and the presence of PGLYRP1 in the pellets was analyzed by Western blot. As can be seen from **Figure 2A**, PGLYRP1 was bound by both live and fixed *E. coli*. Judging by the intensity of the PGLYRP1 bands in the input (lane 2) and bound by bacteria fractions (lane 4), all PGLYRP1 was bound. Since at a ratio of 4∙10^6^ PGLYRP1 monomers per cell was used in the experiment, it follows that a single cell can bind very large amounts of PGLYRP1.

PGLYRP1 also bound to fixed *L. monocytogenes*, *Staphylococcus aureus*, and *Streptococcus pneumoniae* cells (**Figure 2B**). To access the stoichiometry of binding, PGLYRP1 was incubated with various amounts of fixed *L. monocytogenes* cells and its presence in bacterial pellets or the supernatants was determined. The results, shown in **Figure 2C**, showed that almost all PGLYRP1 was cell-bound even at a 10^10^:1 protein molecules per number of cells ratio. We conclude that recombinant PGLYRP1 can bind to various bacteria and there are multiple PGLYRP1 binding sites present at the cell surface.

### PGLYRP1 Has no Bacteriostatic or Bactericidal Activity Towards *L. monocytogenes*

**Figure 3:**
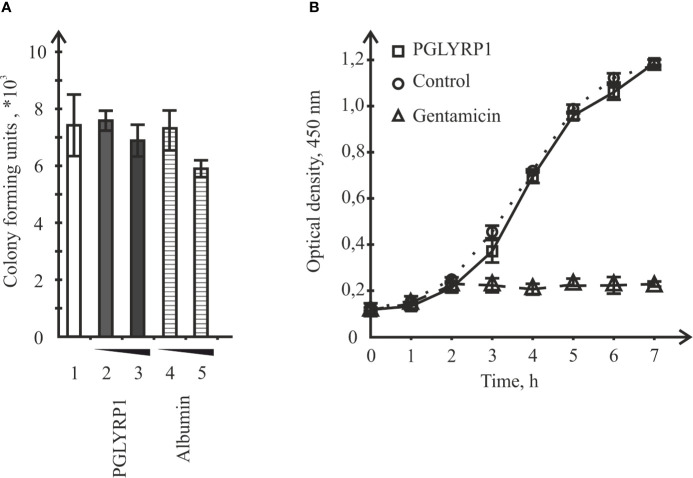
Incubation with recombinant PGLYRP1 has no effect on *L. monocytogenes* viability or growth rate. **(A)** The recombinant PGLYRP1 was incubated for 1 h with live *L. monocytogenes* cells at two concentrations, [5 µg/ml (2) and 2 µg/ml (3)]. Next, bacteria were plated and colony forming units were calculated after overnight growth. Incubation with same concentrations of albumin served as a control. Mean values and standard deviations from three independent experiments are shown. **(B)** Growth of *L. monocytogenes* cultures in the absence or in the presence of 20 µg/ml of recombinant PGLYRP1. Gentamicin (100 µg/ml) added at the 2-h time point served as a positive control. Mean values and standard deviations from three independent experiments are shown.

To check if recombinant human PGLYRP1 exhibited bacteriostatic or bactericidal activity, its effect on colony formation by *L. monocytogenes* was tested. Bacteria (~8.6×10^6^ CFU) were incubated for an hour in the presence of 20 µg/ml or 5 µg/ml of PGLYRP1, corresponding to 6∙10^6^:1 and 1.5∙10^6^:1 protein-cell ratio, respectively. As controls, cells were incubated with recombinant human serum albumin at same concentrations, or without any added protein. The number of colony forming units was next assessed. Comparison of CFU numbers did not reveal any significant effect of PGLYRP1 (**Figure 3A**). The effect of PGLYRP1 on the growth of logarithmic *L. monocytogenes* culture was also monitored. Growth curves in the presence or in the absence of PGLYRP1 (20 µg/ml) were identical (**Figure 3B**), while gentamicin (100 µg/ml) readily inhibited growth, as expected. We conclude that at the conditions of the experiment, PGLYRP1 does not affect *L. monocytogenes* growth.

### PGLYRP1 Has a Small Stimulatory Effect on Phagocytosis of Fixed Bacteria

**Figure 4:**
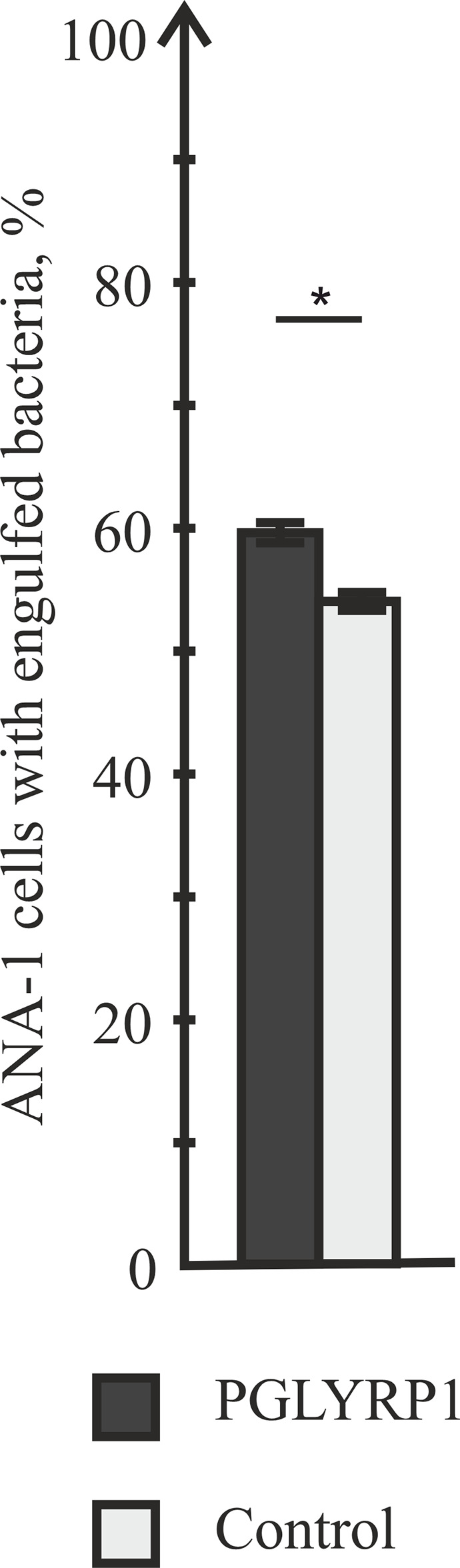
PGLYRP1 marginally affects the phagocytic activity of ANA-1 cells. ANA-1 cells were incubated for 1 h with fixed Cy3-labelled *L. monocytogenes* cells in the presence or in the absence of PGLYRP1 and analysed by flow cytometry. Bars represent percent of ANA-1 cells with engulfed bacteria. Mean values and standard deviations from three independent experiments are shown. *p ≤ 0.05.

Data presented so far show that PGLYRP1 is a secreted protein that effectively interacts with bacterial cell wall but has no antibacterial effect. We hypothesized that PGLYRP1 could function as an opsonin, enabling the immune system to recognize bacterial pathogens more effectively. Therefore, we tested whether interaction of bacteria with PGLYRP1 affected the ability of macrophages to phagocytize them. For this purpose, fixed *L. monocytogenes* labeled with the Cy3 dye (see *Materials and Methods*) were used. ANA-1 macrophage-like mouse cells were cultivated with fixed bacteria (at a ratio of five bacteria per ANA-1 cell). Prior to the addition to ANA-1 cultures, bacteria were either incubated with PGLYRP1 (at a ratio of 4∙10^6^:1 PGLYRP1 monomers per bacterial cell) or with PBS. The effect of incubation with PGLYRP1 on phagocytosis of fixed *L. monocytogenes*-Cy3 was next assayed by flow cytometry. As can be seen from **Figure 4**, PGLYRP1 treatment led to modest, though statistically significant increase of bacteria inside the ANA-1 cells (**Figure 4**).

### Recombinant Human PGLYRP1 Strongly Inhibits Intracellular Survival of *L. monocytogenes*

**Figure 5:**
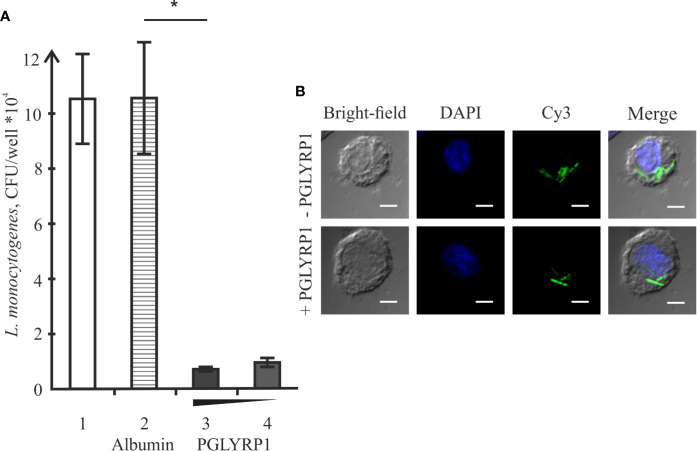
Recombinant PGLYRP1 inhibits intracellular survival of *L. monocytogenes* in ANA-1 cells. **(A)** ANA-1 cells were incubated for 1 h with live *L. monocytogenes* (~100 bacteria per ANA-1 cell) in the presence of PGLYRP1, albumin control, or without any additions. External bacteria were killed by gentamicin, ANA-1 cells were lysed and internalized bacteria were plated for colony forming unit calculation. CFUs obtained after incubation in the absence of added proteins (1), in the presence of albumin (2), and 20 (3) or 10 (4) µg/ml PGLYRP1 are shown. Mean values and standard deviations from three independent experiments are presented. **(B)** Confocal microscopy images of ANA-1 cells and internalized *L. monocytogenes* in the presence or in the absence of PGLYRP1. The nucleus of ANA-1 cell is labeled with DAPI (blue) and bacteria are labelled with Cy3 (green). The scale bars correspond to 10 µm. *p ≤ 0.05.

*L. monocytogenes* is a pathogen that relies on intracellular survival and proliferation inside eukaryotic cells to escape the immune response. We studied the effect of PGLYRP1 on bacterial survival in an *in vitro* infection model. The adhesion culture of ANA-1 cells was infected with *L. monocytogenes* at a multiplicity of 100. Parallel infections were carried in the absence or in the presence of PGLYRP1 at a ~10^9^/10^8^:1 ratio of protein to bacterial cells. The number of viable bacteria that remained inside was determined by counting the number of colonies formed. As can be seen from **Figure 5A**, binding of PGLYRP1 resulted in a nearly 10-fold decrease in the amounts of viable bacteria 2 h after internalization. Combined with the results showing the lack of bacteriostatic or bactericidal activity towards *L. monocytogenes* (**Figure 3**), our data strongly support a suggestion that PGLYRP1 restricts *L. monocytogenes* cells by affecting their viability inside the immune cells. Live fluorescent microscopy failed to reveal differences in morphology or intracellular localization of *L. monocytogenes* cells with or without PGLYRP1 treatment 2 h after internalization (**Figure 5B**). While tentative, the result seems to suggest that PGLYRP1 does not affect the intracellular fate of *L. monocytogenes* by, for example, promoting the fusion of phagosomes with lysosomes.

**Figure 6:**
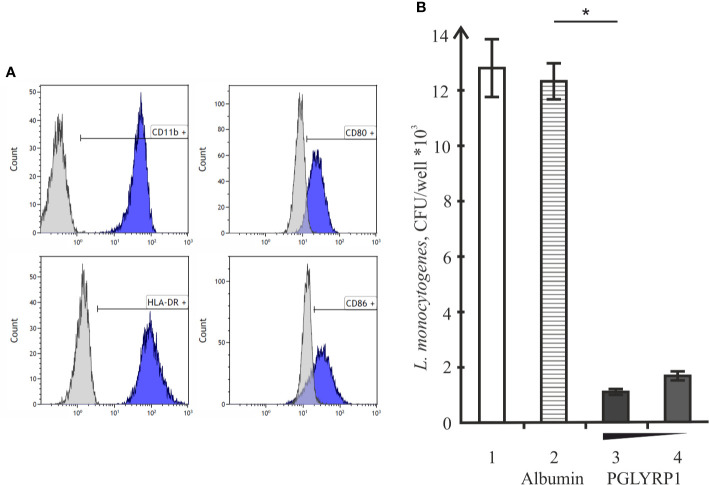
Recombinant PGLYRP1 inhibits intracellular survival of *L. monocytogenes* in human macrophages. **(A)** Flow cytometry analysis of CD11b, CD80, CD86, and HLA-DR markers expression on the surface of monocyte-derived cells. Histograms show the presence of labeled surface markers, grey lines depict isotype controls. **(B)** Monocyte-derived human macrophages were incubated for 1 h with live *L. monocytogenes* (~100 bacteria per macrophage) in the presence of PGLYRP1, albumin control, or without any additions. External bacteria were killed by gentamicin, macrophages were lysed and internalized bacteria were plated for colony forming unit calculation. CFUs obtained after incubation in the absence of added proteins (1), in the presence of albumin (2), and 20 (3) or 10 (4) µg/ml PGLYRP1 are shown. Mean values and standard deviations from three independent experiments are presented. *p ≤ 0.05.

To determine whether PGLYRP1 also impacts *L. monocytogenes* survival inside human cells, human monocyte-derived macrophages were used. Based on flow cytometric analysis, monocyte-derived cells had CD11b, CD80, CD86, and HLA-DR - markers expressed on the M1 macrophages - on their surface (**Figure 6A**). Cells were infected with *L. monocytogenes* at a multiplicity of 100. Parallel infections were carried out in the absence or in the presence of PGLYRP1 at a ~10^9^/10^8^:1 ratio of protein to bacterial cells. As can be seen from **Figure 6B**, the PGLYRP1 treatment of *L. monocytogenes* affected bacterial survival inside human cells as in the case of mouse ANA-1 cells: a 10-fold decrease of colony forming units was obtained when bacteria were recovered from human macrophages after prior PGLYRP1 treatment.

### PGLYRP1 Potentiates Cellular Response to an Activating Stimulus

**Figure 7:**
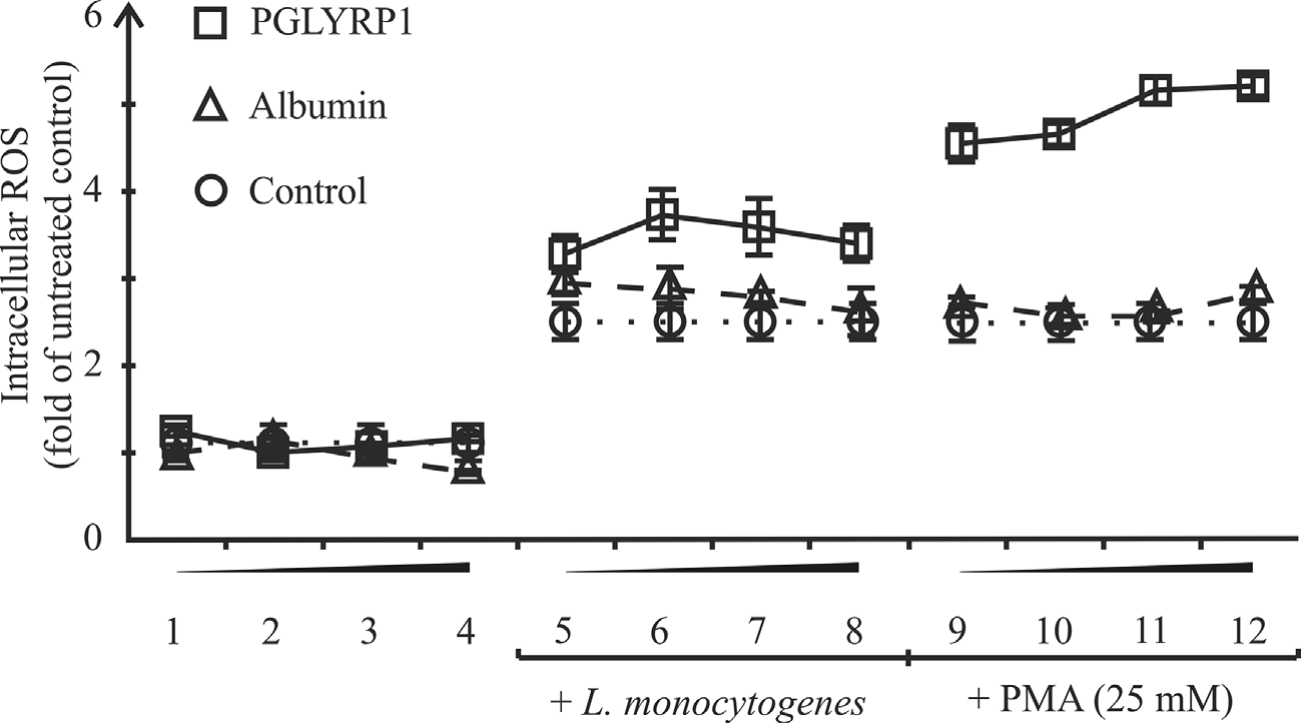
Recombinant PGLYRP1 potentiates oxidative burst response of ANA-1 cells to the PMA stimulus. ANA-1 cells were incubated for 1 h in the presence of different concentrations of PGLYRP1 [0.01 µg/ml (1, 5, 9); 0.1 µg/ml (2, 6, 10); 1 µg/ml (3, 7, 11), or 5 µg/ml (4, 8, 12)], or with same concentrations of albumin, or without any added protein. Where indicated, fixed *L. monocytogenes* or 25 mM PMA was added for oxidative burst response induction. The results were normalized to the ROS signal from untreated ANA-1. Mean values and standard deviations from three independent experiments are shown.

One of the mechanisms of killing intracellular bacteria by macrophages is the generation of reactive oxygen species (ROS). In order to test if PGLYRP1 could stimulate ROS production in macrophages, ANA-1 cells were incubated with fixed *L. monocytogenes* or phorbol-12-myristate-13-acetate (PMA), an activator of oxidative burst, either in the presence of PGLYRP1 or in the presence of albumin, which was not expected to affect oxidative burst. As can be seen from **Figure 7** PGLYRP1 by itself had no effect on ROS production compared to albumin or PBS. PMA increased the ROS production level (in the presence of albumin or PBS) ~3 fold compared to basal level, as expected. Interestingly, the presence of both PGLYRP1 and PMA increased ROS production further, ~5 times compared to the basal level. However, in the presence of *L. monocytogenes* PGLYRP1 (at a ratio of 2∙10^6^:1 PGLYRP1 monomers per bacterial cell) triggered only a marginal (though reproducible) increase of intracellular ROS response compared to albumin. We conclude that, PGLYRP1 alone is not sufficient for increased ROS production, though it potentiates the stimulatory effect of PMA through an unknown mechanism and may slightly increase ROS production by internalized bacteria.

### PGLYRP1 Potentiates IL-6 Cytokine Secretion

**Figure 8:**
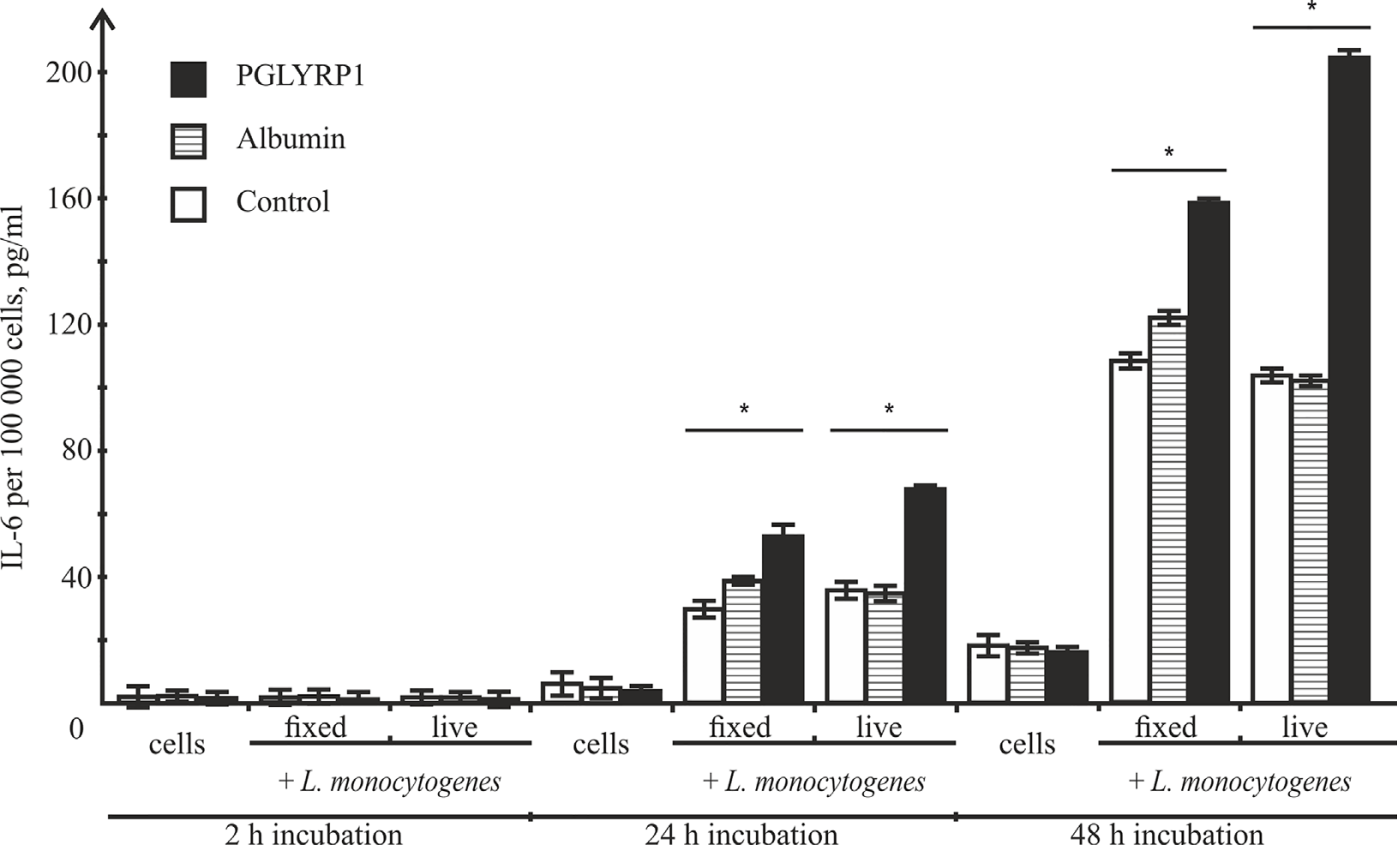
Recombinant PGLYRP1 increases IL-6 production by ANA-1 cells after incubation with *L. monocytogenes*. ANA-1 cells were incubated with fixed (MOI=100) or live (MOI=5) bacteria in the absence or in the presence of 5 µg/ml PGLYRP1 or albumin. Culture supernatants were analysed by ELISA for the presence of IL-6 after 2-, 24-, and 48-h incubation. The results are normalized on 100,000 ANA-1 cells. Mean values and standard deviations from three independent experiments are shown. *p ≤ 0.05.

After infection, activated macrophages with the proinflammatory phenotype produce a number of cytokines including IL-6, which drive inflammatory and anti-microbial responses (Wynn et al., 2013) affecting the spread of bacterial infections in the organism. To determine the effect of PGLYRP1 or PGLYRP1 treatment of bacteria on cytokine secretion, ANA-1 cells were incubated with *L. monocytogenes* (~100 fixed or ~5 live bacteria per ANA-1 cell) in the presence of PGLYRP1 or albumin at a ~10^6^/10^7^:1 protein molecules per bacteria cell ratio. As controls, ANA-1 cells were incubated with PGLYRP1, albumin or PBS only. After 2, 24, and 48-h incubation, the culture supernatants were analyzed by ELISA for the presence of IL-6. As can be seen from **Figure 8**, after 2 h there was no IL-6 in the supernatants. After 24-h incubation, the treatment with PGLYRP1 enhanced secretion of IL-6 in response to *L. monocytogenes* while PGLYRP1 alone had no such effect. An event stronger effect was observed 48 h after the addition of *L. monocytogenes*. The stimulatory effect was more pronounced with live that with fixed bacterial cells.

## Discussion

Peptidoglycan recognition proteins comprise an evolutionarily conserved protein family characterized by the presence of a protein domain highly homologous to the phage lysozyme. Although phages use their lysozyme for direct destruction of the bacterial cell wall by the amidase activity, the peptidoglycan binding domains of PGRPs do not always possess this activity. In this study, we examined the smallest of human PGRPs, PGLYRP1. This protein essentially consists of just the domain homologous to lysozyme and does not exhibit the amidase activity (Wang et al., 2003). In the fruit fly, PGLYRP1 ortholog PGRP-SA plays a significant role in the recognition of Gram-positive bacteria by the innate immune system. High evolutionary conservation implies that mammalian PGLYRP1 also plays an important role in antibacterial immune defense mechanisms. Some authors suggested that PGLYRP1 exhibits direct bacteriostatic or bactericidal effects and can synergize with other effector molecules (Ju et al., 2005). Others revealed that PGLYRP1 has a low direct antibacterial effect but can act through activation of the macrophage response to peptidoglycan/PGLYRP1 complexes (De Marzi et al., 2015). Here, we demonstrated that recombinant human PGLYRP1 protects macrophages and the macrophage-derived cell line ANA-1 from *L. monocytogenes* infection *via* enhancement of intracellular mechanisms of bacterial killing, rather than by direct bactericidal action. Eukaryotic expression system based on the CHO-S cell line was used for production of full-size recombinant PGLYRP1, which was purified from the culture medium by affinity chromatography. The advantage of this approach is the avoidance of contamination of the purified recombinant protein with bacterial cell wall components, which can interfere with its functional effects. Analysis of the purified recombinant PGLYRP1 confirmed the formation of a PGLYRP1 homodimer, as was reported previously (Kiselev et al., 1998; Lu et al., 2006). This observation evidenced the correct folding of the recombinant PGLYRP1, which was further confirmed by its ability to bind to bacterial cell walls. Using a direct binding assay, we demonstrated that recombinant PGLYRP1 efficiently interacted with Gram-positive and Gram-negative bacteria, both live and fixed. Since the cell walls of Gram-positive and Gram-negative bacteria are very different, PGLYRP1 likely interacts with them through different binding sites, as was also suggested for bovine PGRP-S (Tydell et al., 2006). Since direct bactericidal effect of PGLYRP1 is a subject of some controversy, the effect of the recombinant protein on colony formation and growth of *L. monocytogenes* was studied. Our results showed that the binding of PGLYRP1 to bacterial cells does not cause profound bactericidal or bacteriostatic effects, at least at the examined concentrations.

If PGLYRP1 is an evolutionarily conserved protein, which recognizes the bacterial cell wall effectively, but does not mediate direct bacterial killing, what can be its role in immunity? One of the possible explanations is that soluble PGLYRP1 can serve as an opsonin, which makes bacteria more visible to innate immunity mechanisms. Indeed, the treatment of dead *L. monocytogenes* with PGLYRP1 resulted in slightly increased amount of bacteria engulfed by the macrophages. Our observation that PGLYRP1 enhanced phagocytosis of bacteria was similar to that described for the THP-1 cell line (De Marzi et al., 2015). However, since *L. monocytogenes* can use phagocytosis to penetrate macrophages and subsequently invade them to escape the immune response, increased internalization in the presence of PGLYRP1 could actually be detrimental to the host. For this reason, it was important to test the intracellular survival of *L. monocytogenes* after the engulfment. To address this question, the number of intracellular living bacteria after the PGLYRP1 treatment was studied using *in vitro* infection of the ANA-1 cell line. We found that the PGLYRP1 treatment decreased, almost 10-fold, the amount of living bacteria inside the ANA-1 cells after infection, suggesting that PGLYRP1 activates more effective intracellular killing of *L. monocytogenes*. A similar effect was observed for PGRP-L/Tag-L, another member of the PGRP family (Kibardin et al., 2006). Though PGLYRP1 was implicated in intracellular bacterial elimination earlier (Dziarski et al., 2003; Osanai et al., 2011) in the present study we demonstrate that the treatment with PGLYRP1 protects the ANA-1 cells and human macrophages against *L. monocytogenes* infection by increased bacterial killing specifically after phagocytosis. Interestingly, in the case of PGLYRP1, the protection against *L. monocytogenes* was due to killing of bacteria in eukaryotic cell, whereas for PGRP-L/Tag-L, direct killing of bacteria through the amidase activity of its PGRP domain was suggested as the mechanism of action.

One of the mechanisms of intracellular bacterial killing by macrophages is the induction of oxidative burst. PGLYRP1 treatment of ANA-1 cells did not stimulate oxidative burst, whereas pretreatment with PGLYRP1 potentiated subsequent activation of oxidative burst by PMA. However, compared to control, there were no significant differences in oxidative burst of cells after treatment with fixed *L. monocytogenes*. The PGLYRP1-treated ANA-1 cells demonstrated a significantly higher response to the PMA stimulus than cells treated with control protein but, overall, this mechanism is unlikely to explain the considerable difference in intracellular survival of PGLYRP1-treated *L. monocytogenes*.

After exposure to a bacterial stimulus, macrophages secrete different proinflammatory cytokines, such as TNF-α, IL-1, IL-6, IL-8, and IL-12 (Duque and Descoteaux, 2014). In our study we show that the macrophage-derived cell line ANA-1 started to produce proinflammatory cytokine IL-6 after incubation with bacteria, and that co-stimulation with PGLYRP1 potentiated the IL-6 production level. A similar effect was described for THP-1 cells (macrophage cell line), where secretion of proinflammatory cytokines TNF-α, IL-8, and IL-12 was induced by PGLYRP1/peptidoglycan complexes (De Marzi et al., 2015). These observations demonstrate that co-stimulation with PGLYRP1 may potentiate the proinflammatory macrophage response induced by bacteria and/or their components. However, since increased IL-6 production was detected only 24 h after the addition of bacteria, while the death of internalized bacterial cells occurred after just 2 h, increased proinflammatory cytokine production is unlikely to be the cause of PGLIRP1 mediated intracellular bacterial death.

Our data indicate that human PGLYRP1 potentiates proinflammatory response in mouse macrophages after bacterial exposure. Human PGLYRP1 also protected mouse macrophages from *L. monocytogenes* infection by enhancing intracellular bacterial killing and subsequent signal transmission about the inflammation. These two effects are very different but both of them are aimed at prevention of spreading of disease. Intracellular bacteria killing and attraction of components of adaptive immunity by proinflammatory cytokines production may be used as a good start for therapy development against intracellular pathogens.

## Data Availability Statement

The raw data supporting the conclusions of this article will be made available by the authors, without undue reservation.

## Ethics Statement

The studies involving human participants were reviewed and approved by Independent Ethics Committee of Dmitry Rogachev National Medical Research Center Of Pediatric Hematology, Oncology and Immunology. The patients/participants provided their written informed consent to participate in this study.

## Author Contributions

DS, KS, AK, GG, and SL designed the study. DS performed the experiments. AP and AK contributed protein production and purification. EL contributed flow cytometry analysis. ES and SE contributed bacterial analysis. SO contributed confocal microscopy. DS, AK, KS, NG, and GG analyzed experimental data and drafted the manuscript. AP, GG, and SL revised the manuscript. All authors contributed to the article and approved the submitted version.

## Funding

We thank the support of Skolkovo Institute of Science and Technology for PhD fellowship to DS.

## Conflict of Interest

The authors declare that the research was conducted in the absence of any commercial or financial relationships that could be construed as a potential conflict of interest.
